# Associations between *ZNF676*, *CTC*1 Gene Polymorphisms and Relative Leukocyte Telomere Length with Myopia and Its Degree

**DOI:** 10.3390/biomedicines12030538

**Published:** 2024-02-28

**Authors:** Monika Duseikaite, Alvita Vilkeviciute, Edita Kunceviciene, Greta Gedvilaite, Loresa Kriauciuniene, Rasa Liutkeviciene

**Affiliations:** 1 Laboratory of Ophthalmology, Institute of Neuroscience, Lithuanian University of Health Sciences, Eivenių Street 2, LT-50161 Kaunas, Lithuania; alvita.vilkeviciute@lsmuni.lt (A.V.); greta.gedvilaite@lsmuni.lt (G.G.); loresa.kriauciuniene@lsmu.lt (L.K.); rasa.liutkeviciene@lsmu.lt (R.L.); 2 Faculty of Pharmacy, Lithuanian University of Health Sciences, Sukilėlių pr. 13, LT-50166 Kaunas, Lithuania; 3 Faculty of Animal Science, Institute of Biology Systems and Genetic Research, Lithuanian University of Health Sciences, Eiveniu Street 4, LT-50161 Kaunas, Lithuania; edita.kunceviciene@lsmu.lt

**Keywords:** relative leukocyte telomere length, myopia, *ZNF676*, rs412658, *CTC1*, rs3027234

## Abstract

Background: The interaction between environmental and genetic factors that influence eye growth, regulated by vision, contributes to the development and progression of myopia. This dynamic interaction significantly contributes to the multifaceted development and progression of myopia, a prevalent ocular condition. Our study delves into the associations between *ZNF676* and *CTC1* gene polymorphisms and their impact on the relative leukocyte telomere length (relative LTL) in myopia, as well as its degree. By unravelling these underpinnings in conjunction with environmental influences, we aim to enhance our understanding of the complex mechanisms that drive the onset and severity of myopia. Methods: This study included patients with myopia and ophthalmologically healthy subjects. DNA was extracted from peripheral venous blood by the salting out method. Genotyping of *ZNF676* rs412658 and *CTC1* rs3027234, as well as the measurement of relative LTL, were conducted using a real-time polymerase chain reaction method (RT-PCR). The data obtained were statistically analyzed using the “IBM SPSS Statistics 29.0” software program. Results: The results show that myopic patients who are homozygous for the rs3027234 rare allele genotype of the *CTC1* gene have statistically significantly shorter relative LTL compared to patients with the CC and CT genotypes. Also, men with the *CTC1* rs3027234 TT genotype have statistically significantly longer leukocyte telomeres than women with the same genotype. The respective median (IQR) of the relative LTL for women and men is 0.280 (0.463) vs. 0.696 (0.440), with a *p*-value of 0.027. The myopia group with the *ZNF676* rs412658 CC genotype has statistically significantly shorter leukocyte telomeres than the control group with the same genotype (age ≤ 29), and the *p*-value is 0.011. Also, the myopia group with the *ZNF676* rs412658 CT and *CTC1* rs3027234 CT genotypes have statistically significantly longer leukocyte telomeres than the control group with the same genotypes (age > 29), with *p*-values that are, respectively, 0.016 and 0.012. The evaluation of the genotype distributions of the polymorphisms in the myopia patients showed that *ZNF676* rs412658 CT genotype carriers have 4-fold decreased odds of high myopia occurrence (OR = 0.250; CI: 0.076–0.826; *p* = 0.023). Also, the evaluation of the allele distributions of the polymorphism under the additive genetic model in the myopia group showed that the *ZNF676* rs412658 T allele was associated with similar odds of high myopia (OR = 0.269; 95% CI: 0.090–0.807; *p* = 0.019). The comprehensive *p*-value, assessing the relative LTL of subjects across the different levels of myopia, signifies a statistical difference in the relative LTL among individuals with varying degrees of myopia. There was a statistically significant difference in relative LTL between mild and moderate myopia degrees (0.819 (1.983) vs. 0.083 (0.930), *p* = 0.007). Conclusions: *CTC1* rs3027234 TT may be considered a protective genotype for telomere shortening in men, while the overall telomere shortening might be linked to the worse myopia degree. The *ZNF676* rs412658 T allele may protect against a high myopia occurrence.

## 1. Introduction

Myopia is a common refractive error that is characterized by an optical condition in which the parallel light rays entering the eye converge in front of the retina [1]. Myopia is usually caused by an enlarged intraocular chamber, an elongated eyeball shape, or excessive power of the optical system (e.g., an excessively prominent cornea) [2]. Myopia is a significant risk factor for other eye diseases, including cataracts, glaucoma, retinal detachment, and age-related macular degeneration [3]. Given the complications of pathological myopia and other severe disease-related conditions, myopia negatively impacts self-perception, job/activity choice, and eye health. It is also one of the leading causes of blindness worldwide [4]. Myopia’s development and progression arise from the intricate interplay of environmental and genetic factors that regulate eye growth and are influenced by vision [5]. The regulatory mechanisms no longer establish a connection between the growth and the development of the eye’s optical components [6]. 

The authors anticipate that by the year 2050, there will be an estimated 4758 million individuals who are affected by myopia, constituting approximately 49.8% of the global population, with a confidence interval of 43.4% to 55.7% (ranging from 3620 to 6056 million). Additionally, the authors project that 938 million people will experience high myopia, accounting for 9.8% of the world’s population, with a confidence interval of 5.7% to 19.4% (ranging from 479 to 2104 million) [7]. Environmental factors play a decisive role in the development of myopia. The effects of the gene–environment interaction on the etiology of myopia are still controversial, and the results of different studies are contradictory [8]. The implicated genes seem to play roles in synaptic transmission, cell adhesion, calcium ion binding, cation channel activity, and the functioning of the plasma membrane [9]. 

Telomeres, highly conserved hexameric 5′-TTAGGG-3′ repeat DNA sequences, are nucleoprotein structures at each chromosome end that maintain genome stability [10,11]. Such repeats usually contain three or more guanine groups, and the chain containing them, called the enriched G-tail, always forms the 3′ end of the chromosome [12,13]. The telomere is supported by the enzyme telomerase, a ribonucleoprotein complex consisting of a catalytic subunit of telomerase reverse transcriptase (TERT), which synthesizes new telomeric repeats by replication of the telomerase RNA component (TERC) [14]. The shelterin complex largely determines the presence and action of telomere-associated proteins in the telomere sequence. This complex consists of six specialized proteins: (1) telomere repeat-binding factor 1 (TERF1); (2) telomere repeat-binding factor 2 (TERF2); (3) TERF1-interacting nuclear factor 2 (TINF2); (4) telomere protection protein (POT1); (5) tripeptidyl peptidase (TPP1); and (6) TERF2-interacting protein 1 (TERF2IP) [15]. When telomeres are shortened below a crucial threshold, the shelterin complex loses its ability to bind to the telomeric sequence, leading to an inability to block the chromosome end effectively. Consequently, the primary limiting factor for telomere function is its length [16]. Data show that the leukocyte telomere length (LTL) decreases with age and is influenced by gender and lifestyle factors such as obesity, insulin resistance, cigarette smoking, psychological stress, and low socioeconomic status [17]. In addition, a shorter LTL is associated with an increased risk of many chronic diseases such as cardiovascular disease, respiratory disease, type 2 diabetes, liver disease, metabolic syndrome, and neurodegenerative diseases [18,19]. A compilation of studies spanning from 2010 to 2019 focused on experimental investigations involving samples from patients who were diagnosed with hematological malignancies, detailing the reported telomere lengths. While cancer is commonly associated with abnormal telomere shortening, hematological neoplasms exhibit persistent telomere shortening, even following telomerase reactivation [20]. The shortening of telomeres, coupled with chromosomal mutations and the reactivation of telomerase, could potentially serve as predisposing factors for the onset of hematological neoplasms like myelodysplastic syndrome and various forms of leukemia [21]. In hematological malignancies, the presence of telomere shortening is linked with significant genomic rearrangements and DNA damage, phenomena that are absent in cells with longer telomeres. This insight underscores the critical importance of studying and analyzing telomere length for improving patient prognosis in clinical practice [22]. 

Genetic factors underlying the leukocyte telomere length may provide information on telomere homeostasis. *ZNF676* and *CTC1* are known to be responsible for telomere homeostasis [23], so it is essential to uncover their influence on telomere length changes. Zinc finger proteins (ZNFs) are one of the most abundant proteins that perform many molecular functions, such as DNA recognition, RNA packaging, transcriptional activation, regulation of apoptosis, protein folding and assembly, and lipid binding [24]. ZNFs’ classification is based on the structure of the zinc finger region. Many proteins containing classical ZNFs regulate gene expression and interact with specific DNA sequences to target genes’ promoter or enhancer regions [25]. Due to the ability to regulate gene expression, ZNF proteins are also involved in many physiological processes, including cell proliferation, differentiation, and apoptosis, thus supporting tissue homeostasis [26]. The ZNF676 protein is encoded by the *ZNF676* gene, located on chromosome 19 (19p12) [27]. Several recent genome-wide association studies (GWASs) have identified single nucleotide polymorphisms (SNPs) related to leukocyte telomere length [24]. One such SNP is *ZNF676* rs412658, which was investigated in our study. Theoretically, *ZNF676* can modify LTL in two ways: first, direct binding to DNA can alter the expression of genes that are involved in telomere support and interact with RNA or proteins to alter the post-translational signaling of these genes. Second, telomeric DNA can fold into the aforementioned G-quadruplex, inhibiting telomerase elongation at the 3′ telomere end [27]. Zinc finger proteins can specifically bind to G-quadruplex DNA, including telomeric DNA, and stabilize the chromosome end [28].

One of the subunits of the human CTC1-STN1-TEN1 (CST) complex that we studied is the *CTC1* (conservative telomere support component 1) gene. The complex of CST proteins consists of three proteins—a telomeric DNA-binding protein, an oligonucleotide/oligosaccharide-binding folding protein 1 (STN1), and a telomere length-regulating protein (TEN1) [29]. The CST complex is responsible for the replication of telomeric DNA, promoting C-chain synthesis, and involved in regulating the telomere length [30,31,32,33]. Disruption of the CST complex results in the fragility of non-telomeric GC-rich repeat sequences, which is observed in the case of chromosome breakdown [34]. Mangin et al., found that the *CTC1* gene polymorphism rs3027234 was associated with short LTL and decreased *CTC1* gene expression. Based on the gene’s position in the sequence (rs3027234 is located in intron 11–12, 132 bp from exon 11), researchers hypothesized that this SNP might disrupt or create a new splice site that reduces gene expression [27]. Therefore, this study aims to investigate the associations between *ZNF676* rs412658 and *CTC1* rs3027234, along with the measurement of relative leukocyte telomere length (LTL) in relation to the occurrence of myopia and its degree.

## 2. Materials and Methods

### 2.1. Ethics Statement

This study was conducted in the Department of Ophthalmology, Hospital of Lithuanian University of Health Sciences and Laboratory of Ophthalmology, Neuroscience Institute, Lithuanian University of Health Sciences. Ethical approval was obtained from the Ethics Committee for Biomedical Research (BE-2-41 and BE-2-48).

### 2.2. Study Populations

The gene polymorphisms *ZNF676* rs412658 and *CTC1* rs3027234 and relative leukocyte telomere length were examined in 100 patients with myopia and 200 control patients. The control group consisted of healthy individuals undergoing routine ophthalmological examinations at the Hospital of the Lithuanian University of Health Sciences Department of Ophthalmology. The selection involved matching for both age and gender with patients diagnosed with myopia. Inclusion criteria for the control group involved participants displaying no ophthalmological eye disorders during the examination and providing informed consent. Exclusion criteria included any pre-existing eye conditions and the use of epileptic and sedative medications.

### 2.3. DNA Extraction and Genotyping

To isolate deoxyribonucleic acid (DNA), blood was drawn in vacuum tubes containing the anticoagulant EDTA. DNA was isolated from peripheral blood white cells by the DNA salting out method. Genotypes of 2 SNPs were determined using TaqMan^®^ genotyping assays (Applied Biosystems, Foster City, CA, USA): C__11463190_10 (rs412658) and C__15770320_10 (rs3027234). This was carried out according to the manufacturer’s instructions using real-time polymerase chain reaction (RT-PCR) method.

### 2.4. Relative Leukocyte Telomere Length Measurement

Relative LTL in peripheral blood was measured using the quantitative real-time PCR (qPCR) method described by Cawthon (2002) [35]. The amounts of telomere DNA fragments and the reference single copy gene—albumin—in the samples were calculated. We performed 2 replicates of the samples to achieve accuracy. Real-time PCR was performed using a Rotor-Gene Q real-time PCR amplifier (QIAGEN, Hilden, Germany) to determine relative LTL. An age-matched sample from the control group was used as reference DNA. DNA isolated from a commercial human cell line 1301 with an extra-long telomere (Sigma Aldrich, St. Louis, MO, USA) was a positive control. The relative (LTL) method, as well as forward and reverse primers, have been described in detail in our previous study [36]. The used primers were ordered from IDT (Integrated DNA Technologies, Inc., Coralville, IA, USA). Relative LTL for each sample was assessed through two distinct qPCR runs. The initial run aimed to identify the cycle threshold (Ct) value for telomere amplification, while the subsequent run was conducted to ascertain the Ct value for control gene amplification [37]. The standard curve in each run consisted of a serial 6-point dilution of the reference DNA pool. The Ct data generated in both runs were used to calculate the relative LTL values for each sample: LTL = 2^−ΔΔCt^ [38].

### 2.5. Statistical Analysis

The results were analyzed using the statistical program “IBM SPSS Statistics 29.0”. The data obtained are presented in absolute numbers (percentages), median values, and interquartile ranges (IQRs). The Mann–Whitney U-test method determined the differences between the two independent groups when the data were not normally distributed. Student’s *t*-test was used in a normal data distribution. We used the nonparametric Kruskal–Wallis test to compare more than two groups. The distributions of polymorphisms of the studied genes in the control group were evaluated according to the Hardy–Weinberg equilibrium [39]. The Pearson Chi-square (χ2) test was used to calculate the genotype distribution, allele frequencies, and statistical reliability parameters for the SNPs *ZNF676* rs412658 and *CTC1* rs3027234. Binary logistic regression was used to assess myopia’s probability of occurrence (PO) by genotype combination based on genetic inheritance. The same analysis was performed for individual myopia groups according to the subjects’ sex, with a PO of 95% confidence interval (CI). The Akaike Information Criterion (AIC) determined the best-fit inheritance model. Statistically, significant differences and correlations were found when the *p*-value < 0.05. 

### 2.6. Limitations

While our study provides valuable insights into telomere dynamics, several limitations warrant consideration. Firstly, although participants were matched for age and gender, variables such as smoking, obesity, and stress disorders, known to influence telomere length, were not specifically accounted for in our analysis. Consequently, the impact of these factors on telomere attrition remains unexplored within our study. Secondly, in our investigation, we conducted an analysis of DNA extracted from venous blood leukocytes. It is important to note that our study did not specifically differentiate between various leukocyte populations or subpopulations. Thirdly, our sample size may limit the generalizability of our findings to broader populations. Future investigations should incorporate comprehensive assessments of lifestyle factors to elucidate the complex interplay between environmental influences and telomere dynamics accurately. Fourthly, myopia can result from a steep cornea with a normal axial length, and this group can behave as normal controls. Despite these limitations, our study underscores the importance of further research to comprehensively understand telomere regulation and its implications for health and disease. 

## 3. Results

### 3.1. ZNF676 rs412658 and CTC1 rs3027234 Determination of Single Nucleotide Polymorphisms and Leukocyte Telomere Length in Healthy Subjects and Myopia Group

During the study, subjects were divided into myopia and control groups. In the myopia group (n = 100), 27 were men (27%), 73 were women (73%), and the age median (IQR) was 26.5 (24). The control group consisted of 200 subjects: 51 males (25.5%) and 149 females (74.5%), with an age median (IQR) of 29 (21). The demographic characteristics of the study are shown in Table S1 of the Supplementary Materials.

The genotypes and allele distributions of *ZNF676* rs412658 and *CTC1* rs3027234 were examined in the myopia group and compared with the control group. The genotype frequencies of the tested polymorphisms were in accordance with the Hardy–Weinberg equilibrium (HWE), and the results are shown in Table 1. The frequency distributions of *ZNF676* rs412658 and *CTC1* rs3027234 alleles and genotypes in healthy subjects and the myopia group showed no statistically significant differences (Supplementary Materials, Table S2). Binary logistic regression analysis also revealed no statistically significant association with myopia (Supplementary Materials, Table S3). 

*ZNF676* rs412658 and *CTC1* rs3027234 genotype and allele distribution were compared by gender. Unfortunately, no statistically significant differences were found (Supplementary Materials, Tables S4 and S5). Binary logistic regression analysis also did not yield statistically significant results when divided by gender (Supplementary Materials, Tables S6 and S7).

**Figure 1:**
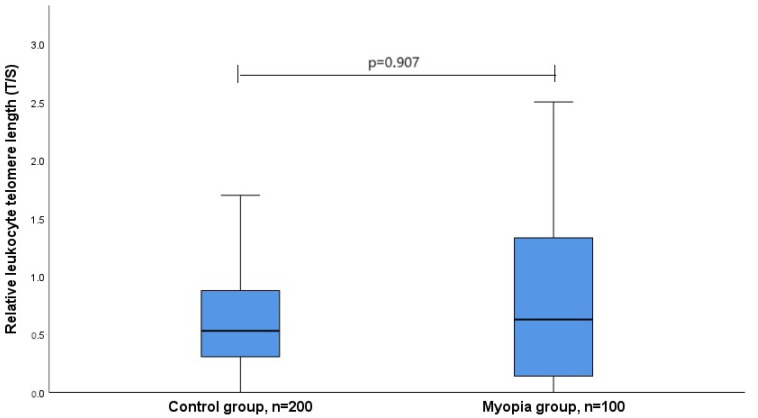
Relative leukocyte telomere length in control and myopia groups. The X-axis shows the study groups, the Y-axis shows the relative leukocyte telomere length, and the blue rectangles divided by the medians denote the quartiles. The lower part is the first quartile, and the upper part is the third quartile; *p*-value is the significance level (differences are considered statistically significant if *p* < 0.05).

Later in the study, the relative LTL in the control and myopia groups were determined. The control group had a maximum relative LTL of 5.01024 and a minimum of 0.00017. In the myopia sample, the maximum relative LTL was 4.78328, and the lowest was 0.00024. When comparing the control and myopia groups, we found that the relative LTL of the myopia group was similar to the control group: the median (IQR) was 0.594 (1.198) vs. 0.529 (0.572), *p* = 0.907. The data are shown in Figure 1.

**Figure 2:**
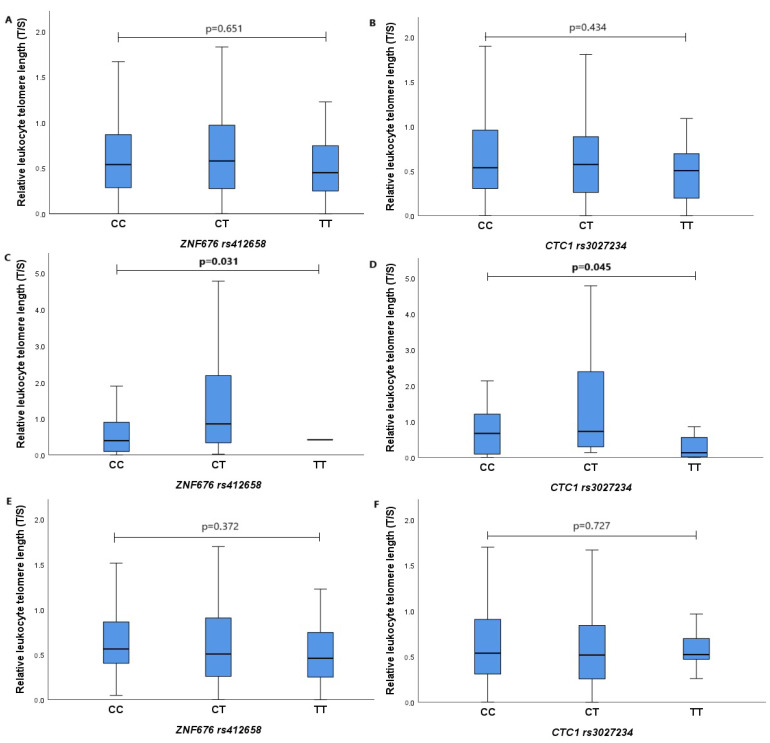
Relative leukocyte telomere length’s association with *ZNF676* rs412658 and *CTC1* rs3027234 gene polymorphisms in subjects. Blue rectangles, divided by medians, denote quartiles. The lower part is the first quartile, and the upper part is the third quartile. The X-axis represents polymorphism genotypes, the Y-axis indicates relative leukocyte telomere length; *p*-value indicates the level of significance (differences are considered statistically significant when *p* < 0.05). (**A**)—the relationship between relative leukocyte telomere length and *ZNF676* rs412658 polymorphism in both groups; (**B**)—the relationship between relative leukocyte telomere length and *CTC1* rs3027234 polymorphism in both groups; (**C**)—the relationship between relative leukocyte telomeres and *ZNF676* rs412658 polymorphism in the myopia group; (**D**)—the relationship between relative leukocyte telomeres and *CTC1* rs3027234 polymorphism in the myopia group; (**E**)—the relationship between relative leukocyte telomeres and *ZNF676* rs412658 polymorphism in the control group; (**F**)—the relationship between relative leukocyte telomeres and *CTC1* rs3027234 polymorphism in the control group.

The associations between the relative LTL and the genotypes of *ZNF676* rs412658 and *CTC1* rs3027234 polymorphisms in different study groups were also analyzed. Figure 2A shows the association between the telomere length and *ZNF676* rs412658 polymorphism in both groups combined. The relative LTL median of the subjects for the CC, CT, and TT genotypes were determined, respectively, as a median (IQR) of 0.538 (0.593) vs. 0.578 (0.702) vs. 0.450 (0.522), with *p* = 0.651. Figure 2B shows the association between the *CTC1* rs3027234 polymorphism and LTL in both groups. The relative LTL of the subjects in the CC, CT, and TT genotype groups were found to be 0.537 (0.668) vs. 0.572 (0.635) vs. 0.504 (0.562), with *p* = 0.434. Figure 2C shows the association between the *ZNF676* rs412658 polymorphism and LTL in the myopia group comparing the CC and CT genotypes, whereas the TT genotype was not detected in this group, with a median (IQR) of 0.394 (0.829) vs. 0.855 (1.972), and *p* = 0.031 (Mann–Whitney U test). Figure 2D shows the association between the *CTC1* rs3027234 polymorphism and the relative LTL in the myopia group, comparing the CC, CT, and TT genotypes, respectively, with the median (IQR): 0.671 (1.14) vs. 0.726 (2.13) vs. 0.131 (0.622); *p* = 0.045. In this group, we also compared the distribution of genotypes under the dominant and recessive genetic models and their influence on LTL: CC vs. CT+TT, *p* = 0.641, and CC+CT vs. TT, *p* = 0.044 (Mann–Whitney U Test). Figure 2E shows the association between the *ZNF676* rs412658 polymorphism and the relative LTL in the control group, comparing the CC, CT, and TT genotypes, with a median (IQR) of 0.562 (0.471) vs. 0.506 (0.658) vs. 0.459 (0.550), and *p* = 0.372. Figure 2F shows the association between the *CTC1* rs3027234 polymorphism and LTL in the control group. The relative LTL medians in the CC, CT, and TT genotype groups were found to be 0.537 (0.609) vs. 0.515 (0.598) vs. 0.521 (0.444), with *p* = 0.727. The results show that myopic patients with the homozygous genotype for the *CTC1* rs3027234 rare allele have a statistically significantly shorter relative LTL compared to patients with the CC and CT genotypes. Figure 2A–F are shown in Figure 2.

**Figure 3:**
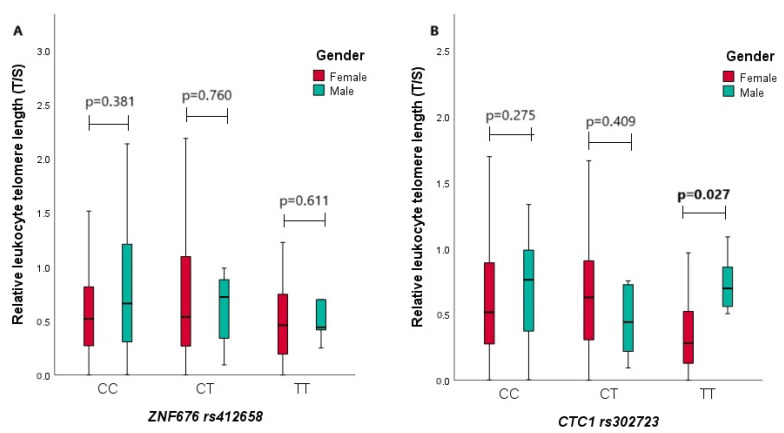
Comparison of relative leukocyte telomere length and genotypes of *ZNF676* rs412658 and *CTC1* rs3027234 gene polymorphisms in male and female groups in all study participants. (**A**,**B**) On the X-axis, the genotypes of *ZNF676* rs412658 and *CTC1* rs3027234 gene polymorphisms are illustrated, with the Y-axis representing the relative leukocyte telomere length. Red and green rectangles, separated by medians, signify quartiles. The lower segment represents the first quartile, and the upper segment represents the third quartile. The *p*-value indicates the level of significance, with differences considered statistically significant when *p* < 0.05.

We also compared the relative LTL of all male and female subjects in the *ZNF676* rs412658 and *CTC1* rs3027234 genotype groups (Figure 3). In Figure 3A,B, the relative LTL is depicted based on genotypes for both women and men. Figure 3A shows the relationship between the relative LTL and the *ZNF676* rs412658 polymorphism concerning gender. From the diagram, it can be observed that there were no statistically significant differences between the relative LTL of men and women in every genotype group. Specifically, the median (IQR) of the relative LTL for women and men are, for the CC genotype, 0.519 (0.548) vs. 0.661 (0.947), for the CT genotype 0.537 (0.849) vs. 0.720 (0.598), and for the TT genotype 0.459 (0.580) vs. 0.440 (1.604), respectively. Figure 3B shows the relationship between the *CTC1* rs3027234 polymorphism genotypes and the relative LTL and gender. The data indicate that men with the *CTC1* rs3027234 TT genotype have statistically significantly longer leukocyte telomeres than women with the same genotype. The respective median (IQR) of relative LTL for women and men is 0.280 (0.463) vs. 0.696 (0.440), with a *p*-value of 0.027.

Similarly, the analysis of the association between the relative LTL and *ZNF676* rs412658 and *CTC1* rs3027234 genotypes with gender was conducted in the myopia group (Supplementary Materials, Figure S1). Notably, the *ZNF676* rs412658 TT genotype was not identified in either males or females. Regarding the *CTC1* rs3027234, only females exhibited the CC genotype, while only males displayed the TT genotype. Upon comprehensive examination of the data, no statistically significant differences in relative LTL were observed between males and females across various genotype groups (*p* > 0.05). However, it is essential to note that a more extensive inclusion of subjects is required for a more precise analysis and results.

**Figure 4:**
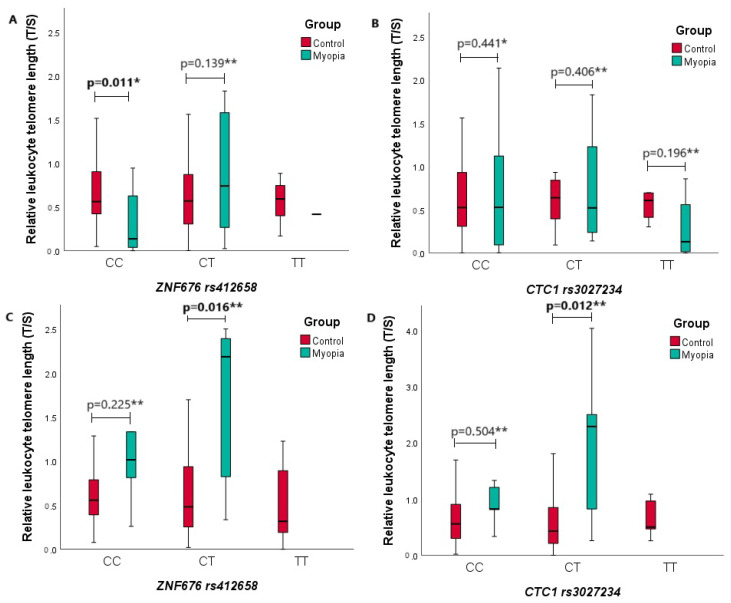
Comparison of relative leukocyte telomere length and genotypes of *ZNF676* rs412658 and *CTC1* rs3027234 gene polymorphisms in two age groups. (**A**,**B**) Groups’ age is less or equal to 29 years old; (**C**,**D**) groups are more than 29 years old. On the X-axis, the genotypes of *ZNF676* rs412658 and *CTC1* rs3027234 gene polymorphisms are illustrated, with the Y-axis representing the relative leukocyte telomere length. Red and green rectangles, separated by medians, signify quartiles. The lower segment represents the first quartile, and the upper segment represents the third quartile. The *p*-value indicates the level of significance, with differences considered statistically significant when *p* < 0.05. *—Mann–Whitney U test was used; **—Student‘s *t*-Test was used.

Continuing the investigation, we compared the relative LTL of the two study groups in the *ZNF676* rs412658 and *CTC1* rs3027234 genotype groups (Figure 4). In Figure 4A,B, the relative LTL is depicted based on the age median for both the control and myopia groups (age ≤ 29). Figure 4A shows the relationship between the relative LTL and the *ZNF676* rs412658 polymorphism based on the defined age group. From the diagram, it can be seen that the myopia group with the *ZNF676* rs412658 CC genotype has statistically significantly shorter leukocyte telomeres than the control group with the same genotype. The respective median (IQR) of the relative LTL for the control and myopia group is 0.562 (0.510) vs. 0.136 (0.651), with a *p*-value of 0.011. Figure 4B shows the relationship between the relative LTL and the *CTC1* rs3027234 polymorphism based on the aforementioned age group. From the diagram, it can be observed that there were no statistically significant differences between the relative LTL of both subject groups in every genotype group. In Figure 4C,D, the relative LTL is depicted based on the age median for both the control and myopia groups (age > 29). The C figureresults show that the myopia group with the *ZNF676* rs412658 CT genotype has statistically significantly longer leukocyte telomeres than the control group with the same genotype. The respective mean (SD) of the relative LTL for the control and myopia group is 0.723 (0.750) vs. 1.648 (0.996), with a *p*-value of 0.016. From Figure 4D, it can be observed that the myopia group with the *CTC1* rs3027234 CT genotype also has statistically significantly longer leukocyte telomeres than the control group with the same genotype. The respective mean (SD) of the relative LTL for the control and myopia group is 0.713 (0.957) vs. 2.033 (1.340), with a *p*-value of 0.012.

### 3.2. ZNF676 rs412658 and CTC1 rs3027234 Gene Single Nucleotide Polymorphisms and Leukocyte Telomere Length Correlations with Myopia Degrees

The degree of myopia is characterized by three degrees: mild, moderate, and high. The evaluation of the genotype distributions of the polymorphisms in the myopia patients showed that *ZNF676* rs412658 CT genotype carriers have 4-fold decreased odds of high myopia occurrence (OR = 0.250; CI: 0.076–0.826; *p* = 0.023) (Table 2). Also, the evaluation of the allele distributions of the polymorphisms in the myopia group showed that the *ZNF676* rs412658 T allele was associated with similarly decreased odds of high myopia (OR = 0.269; 95% CI: 0.090–0.807; *p* = 0.019) (Table 3). 

**Figure 5:**
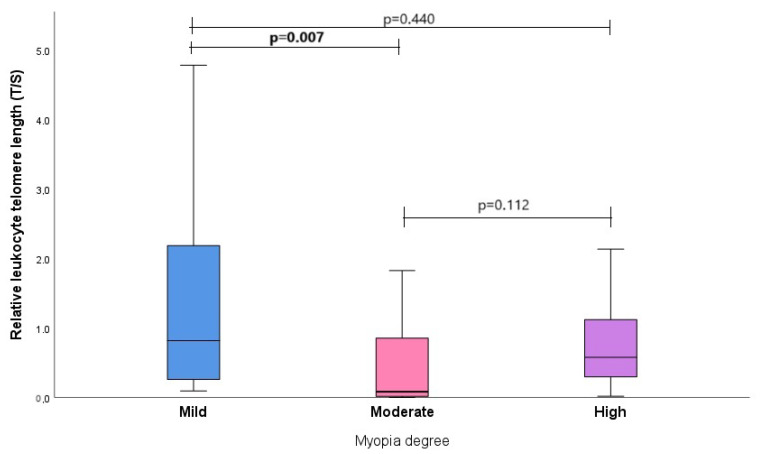
Myopia degree associations with relative leukocyte telomere length. The weak, moderate, and high myopia degrees are illustrated on the X-axis, with the Y-axis representing the relative leukocyte telomere length. Blue, pink, and purple rectangles, separated by medians, signify quartiles. The lower segment represents the first quartile, and the upper segment represents the third quartile. The *p*-value indicates the level of significance, with differences considered statistically significant when *p* < 0.05.

Continuing the study, the relative LTL was also compared in the study subjects concerning the degree of myopia. Subjects’ median (IQR) for mild, moderate, and high myopia was 0.819 (1.983) vs. 0.083 (0.930) vs. 0.578 (0.927), respectively. The comprehensive *p*-value, assessing the relative LTL of subjects across the three levels of myopia, was determined to be 0.027. This signifies a statistical difference in the relative LTL among individuals with varying degrees of myopia. The relative LTL was also compared between different degrees of myopia (Figure 5). In Figure 5, it can be seen that there was a statistically significant difference in the relative LTL between mild and moderate myopia degrees (0.819 (1.983) vs. 0.083 (0.930), *p* = 0.007). 

We also compared the relative LTL and the distribution of genotypes in different degrees of myopia. The medians of the distribution of genotypes in different degrees of myopia are shown in Supplementary Materials, Table S8. It should be noted that the *ZNF676* rs412658 TT genotype was not detected in the study subjects. A comparison of the relative LTL and genotype distributions across different degrees of myopia did not reveal any statistically significant results, as in all comparisons, *p* > 0.05. The figures are shown in Supplementary Materials, Figures S2 and S3.

## 4. Discussion

Myopia has emerged as an epidemic in numerous European and particularly Asian nations. Within this geographical region, the occurrence of myopia among young adults who have undergone 12 to 13 years of schooling has escalated from 70% to 90%, a significant increase from the 20% to 30% that was observed two or three generations ago [1]. In addition, the prevalence of high and potentially pathological myopia of more than −6D has increased [40,41,42]. According to some projections, almost 50% of the world’s population could be myopic by 2050, with about 10% being highly myopic [7].

Genetic experts [43,44] concur that the notable surge in myopia prevalence in these regions cannot be solely attributed to genetic factors. While acknowledging the role of genetics, it is evident that the escalating rates of myopia are inconsistent with a predominantly genetic etiology. Genetic variations have been identified as contributing to a minimum of 12% of the variability in the mean spherical equivalent refraction (SER) in contemporary populations of European descent [44] and possibly 30% or more [45]. A comprehensive overview of the evidence supporting the link between genetic factors and myopia can be found in another article within the IMI series [46]. Despite the relatively stable nature of gene pools across generations, alterations in the natural and social environment can undergo more rapid changes [47].

So, the aim of our study was to delve into the associations between *ZNF676* and *CTC1* gene polymorphisms and their impact on the relative LTL in myopia and its degree. By unraveling these underpinnings in conjunction with environmental influences, we aim to enhance our understanding of the complex mechanisms that drive the onset and degree of myopia. The neural retina possesses the longest telomeres among all the examined ocular structures, while the cornea has the shortest. Specifically, the retinal pigment epithelium demonstrates telomeres that are approximately four times shorter than those in the neural retina. Interestingly, there is no observed age-related decrease in telomere length within the retina, nor is there a distinction in telomere lengths between the macular region and the remaining retina [48]. In the course of our investigation, DNA was extracted from the subjects’ peripheral blood, subsequently enabling a comprehensive analysis of the telomere lengths and their discernible relationship with the aforementioned disease.

The association of the relative LTL and the *ZNF676* rs412658 and *CTC1* rs3027234 polymorphisms with the incidence and degree of myopia has not yet been analyzed. However, these two genes play an essential role in regulating and maintaining telomere length [24,29].

Telomere length, measured in lymphocytes (LTL), indicates risk of various cancers [49]. Recent studies have shown that telomere dysfunction may have a dual effect on carcinogenesis: both short and long telomeres can contribute to cancer development. Shortening telomeres can lead to increased chromosomal end fusion, resulting in genome instability and malignant tumor transformation. Interestingly, a higher relative LTL was associated with an increased cancer risk in women, including a risk of breast, ovarian, and endometrial cancers (OR = 1.44; 95% CI: 1.18–1.75, *p* < 0.001) [50]. The association between a high relative LTL and an increased soft tissue carcinoma (SAC) risk was highly significant in women but not in men [45]. In our study, when comparing the study groups, it was found that the myopia group’s relative LTL was similar to the control group: the median (IQR) was 0.594 (1.198) vs. 0.529 (0.572), and *p* = 0.907.

Moreover, we compared the relative LTL in relation to the degree of myopia. The comprehensive *p*-value, assessing the relative LTL of subjects across the three levels of myopia, was determined to be 0.027. This signifies a statistical difference in the relative LTL among individuals with varying degrees of myopia. The relative LTL was also compared between different degrees of myopia, and we found that there was a statistically significant difference in the relative LTL between mild and moderate myopia degrees (0.819 (1.983) vs. 0.083 (0.930), *p* = 0.007). 

The literature indicates that the most common C allele of *ZNF676* rs412658 near the 3′ end of *ZNF676* is associated with a short relative LTL [27]. Analyzing the interactions of *ZNF676* rs412658, Xu and co-researchers found that the influence of the *ZNF676* rs412658 polymorphism was associated with soft tissue sarcoma (SAC) and LTL [50]. In their study, Mangino et al. found that the most common rs412658 allele was associated with a longer relative LTL [27]. In contrast, in our study, we found that each rare T allele of the *ZNF676* rs412658 polymorphism was associated with 4-fold decreased odds of a high myopia degree occurrence (OR = 0.269; 95% CI: 0.090–0.807; *p* = 0.019). Also, myopia patients showed that *ZNF676* rs412658 CT genotype carriers have 4-fold decreased odds of a high myopia degree occurrence (OR = 0.250; CI: 0.076–0.826; *p* = 0.023). In studies analyzing *CTC1* rs3027234, Walsh et al. found that the *CTC1* rs3027234 polymorphism was statistically significantly associated with brain tumor (glioma) development (OR = 1.14; 95% CI: 1.02–1.28; *p* = 0.020) [51]. 

An expression analysis by Mangino and co-authors showed that the rare T allele of *CTC1* rs3027234 was associated with both a short relative LTL and decreased expression of the *CTC1* gene [27]. In addition, the men in our study who had the *CTC1* rs3027234 TT genotype had statistically significantly longer leukocyte telomeres than women with the same genotype. The respective median (IQR) of the relative LTL for women and men was 0.280 (0.463) vs. 0.696 (0.440), with a *p*-value of 0.027. A recent whole-exon sequencing study revealed that *CTC1* missense mutations cause human Coats plus (CP) disease. Interestingly, the white blood cell telomeres of CP patients with multiple heterozygous mutations are very short, suggesting that the telomere support function of these patients is impaired [29]. Some studies show that white blood cell telomeres are longer in women than in men [52]. This may be due to the presence of an estrogen response element in the promoter region of the *TERT* gene, which could affect the expression of this gene and contribute to telomere recovery. It should be noted that telomeric sequences are susceptible to oxidative stress. Importantly, the higher estrogen levels in women cause less reactive oxygen species (ROS) than in men. This hormone reduces ROS production while being a potent antioxidant and a regulator of antioxidant genes [52,53]. 

## 5. Conclusions

Our study revealed that *CTC1* rs3027234 TT may be considered a protective genotype for telomere shortening in men, while the overall telomere shortening might be linked to a high myopia degree compared to patients with a mild myopia degree. On the other hand, the *ZNF676* rs412658 T allele may protect against a high myopia degree occurrence, but further analysis with larger study groups is necessary to confirm these findings.

## Figures and Tables

**Table 1:** Analysis of Hardy–Weinberg equilibrium (HWE) in the control group.

SNP	Allele Frequencies		Genotype Distribution	HWE *p*-Value
*ZNF676* rs412658	0.62 (C)	0.37 (T)	77/97/26	0.596
*CTC1* rs3027234	0.78 (C)	0.22 (T)	128/59/13	0.093

**Table 2:** The associations of *ZNF676* rs412658 and *CTC1* rs3027234 gene polymorphisms with degrees of myopia.

		Genotypes				
*ZNF676* rs412658		CC (%)	CT (%)	TT (%)	*p*-value (CC vs. CT + TT)	OR (95% CI)
Myopia	Mild (n = 66)	27 (22)	33 (22.9)	6 (18.2)	0.728	0.904 (0.513–1.595)
	Moderate (n = 20)	9 (7.3)	10 (6.9)	1 (3)	0.571	0.765 (0.303–1.931)
	High (n = 14)	10 (8.1)	4 (2.8)	-	**0.023**	0.250 (0.076–0.826)
Control	Emmetropia (n = 200)	77 (62.6)	97 (67.4)	26 (78.8)	-	-
*CTC1* rs3027234		CC (%)	CT (%)	TT (%)	*p*-value (CC vs. CT + TT)	OR (95% CI)
Myopia	Mild (n = 66)	34 (18.2)	28 (30.1)	4 (20)	0.073	1.673 (0.953–2.937)
	Moderate (n = 20)	14 (7.5)	3 (3.2)	3 (15)	0.594	0.762 (0.281–2.069)
	High (n = 14)	11 (5.9)	3 (3.2)	-	0.278	0.485 (0.131–1.795)
Control	Emmetropia (n = 200)	128 (68.4)	59 (63.4)	13 (65)	-	-

*p*-value—level of significance; differences were considered statistically significant when *p* < 0.05; OR—capability ratio. Statistically significant values in the tables are in bold.

**Table 3:** The allelic associations of *ZNF676* rs412658 and *CTC1* rs3027234 gene polymorphisms with degrees of myopia.

		Alleles			
*ZNF676* rs412658		RA	RA Frequency (%)	*p*-value	OR (95% CI)
Myopia	Mild (n = 66)	T	45 (34.1)	0.502	0.864 (0.565–1.322)
	Moderate (n = 20)		12 (30)	0.354	0.710 (0.344–1.465)
	High (n = 14)		4 (14.3)	**0.019**	0.269 (0.090–0.807)
Control	Emmetropia (n = 200)		149 (37.3)	-	-
*CTC1* rs3027234					
Myopia	Mild (n = 66)	T	36 (27.3)	0.168	1.361 (0.878–2.110)
	Moderate (n = 20)		9 (22.5)	0.865	1.065 (0.517–2.193)
	High (n = 14)		3 (10.7)	0.218	0.475 (0.146–1.552)
Control	Emmetropia (n = 200)		85 (21.3)	-	-

*p*-value—level of significance; differences were considered statistically significant when *p* < 0.05; OR—capability ratio; RA—rare allele. Statistically significant values in the tables are in bold.

## Data Availability

Data will be sent upon a request.

## Supplementary Materials

The following supporting information can be downloaded at https://www.mdpi.com/article/10.3390/biomedicines12030538/s1: Table S1: Demographic characteristics of the study; Table S2: Frequencies of genotypes and alleles of *ZNF676* rs412658 and *CTC1* rs3027234 in patients with myopia and the control group; Table S3: Binominal logistic regression analysis of *ZNF676* rs412658 and *CTC1* rs3027234 in the control and myopia groups; Table S4: Frequencies of genotypes and alleles of *ZNF676* rs412658 and *CTC1* rs3027234 in female patients with myopia and the control group; Table S5: Binominal logistic regression analysis of *ZNF676* rs412658 and *CTC1* rs3027234 in the female control and myopia groups; Table S6: Frequencies of genotypes and alleles of *ZNF676* rs412658 and *CTC1* rs3027234 in male patients with myopia and the control group; Table S7: Binominal logistic regression analysis of *ZNF676* rs412658 and *CTC1* rs3027234 in the male control and myopia groups; Figure S1: Comparison of relative leukocyte telomere length and genotypes of *ZNF676* rs412658 and *CTC1* rs3027234 gene polymorphisms in female and male myopia patients; Table S8: Medians of the *ZNF676* rs412658 and *CTC1* rs3027234 genotype distributions with telomere length according to myopia degree; Figure S2: *ZNF676* rs412658 genotype associations with relative leukocyte telomere length according to myopia degree; Figure S3: *CTC1* rs3027234 genotype associations with relative leukocyte telomere length according to myopia degree.
