# Supplementary material for: Associations between ZNF676, CTC1 Gene Polymorphisms and Relative Leukocyte Telomere Length with Myopia and Its Degree
Source: Biomedicines. 2024 Feb 28;12(3):538. doi: 10.3390/biomedicines12030538 (PMC10968307; doi:10.3390/biomedicines12030538)
Supplement: Supplementary file 1 [file biomedicines-12-00538-s001.zip › biomedicines-2855333-supplementary.pdf]

Supplementary material

Table S1. Demographic characteristics of the study

Characteristics	Group		*p*-Value
	**Myopia group, n=100**	**Control group, n=200**	
**Male, *n* (%)**	27 (27)	51 (25.5)	0.780
**Female, *n* (%)**	73 (73)	149 (74.5)	
**Age median (IQR)**	26.5 (24)	29 (21)	0.125

IQR – interquartile range.

Table S2. Frequencies of genotypes and alleles of *ZNF676* rs412658 and *CTC1* rs3027234 in patients with myopia and the control group.

Polymorphism	Genotype/ allele	Frequency (%)		
		**Control group, n=200**	**Myopia group, n=100**	***p*-Value**
ZNF676 rs412658	**Genotype**			
	CC	77 (38.5)	46 (46)	0.209
	CT	97 (48.5)	47 (47)	
	TT	26 (13)	7 (7)	
	Total	200 (100)	100 (100)	
	**Allele**			
	C	251 (62.8)	139 (69.5)	0.102
	T	149 (37.2)	61 (30.5)	
CTC1 rs3027234	**Genotype**			
	CC	128 (64)	59 (59)	0.695
	CT	59 (29.5)	34 (34)	
	TT	13 (6.5)	7 (7)	
	Total	200 (100)	100 (100)	
	**Allele**			
	C	315 (78.8)	152 (76)	0.444
	T	85 (21.2)	48 (24)	

p-value – level of significance; differences were considered statistically significant when p<0.05.

Table S3. Binominal logistic regression analysis of *ZNF676* rs412658 and *CTC1* rs3027234 in the control and myopia groups.

Model	Genotype/allele	OR (95% CI)	*p*-Value	AIC
***ZNF676* rs412658**				
Codominant	C/T *vs.* C/C<br>T/T *vs.* C/C	0.811 (0.490 – 1.344)<br>0.451 (0.181 – 1.121)	0.416<br>0.086	382.620
Dominant	C/T+T/T *vs.* C/C	0.735 (0.452 – 1.194)	0.214	382.366
Recessive	T/T *vs.* C/T+C/C	0.504 (0.211 – 1.204)	0.123	381.282
Overdominant	C/T *vs.* C/C+T/T	0.942 (0.582 – 1.523)	0.806	383.848
Additive	T	0.725 (0.498 – 1.056)	0.094	381.045
***CTC1* rs3027234**				
Codominant	C/T *vs.* C/C	1.250 (0.741 – 2.109)	0.402	385.185

	T/T *vs.* C/C	1.168 (0.443 – 3.079)	0.753	
Dominant	C/T+T/T *vs.* C/C	1.235 (0.755 – 2.021)	0.400	383.202
Recessive	T/T *vs.* C/T+C/C	1.083 (0.418 – 2.805)	0.870	383.882
Overdominant	C/T *vs.* C/C+T/T	1.231 (0.737 – 2.057)	0.427	383.282
Additive	T	1.153 (0.785 – 1.694)	0.467	383.383

p-value – level of significance; differences were considered statistically significant when p<0.05; OR – capability ratio; AIC – Akaike.

Table S4. Frequencies of genotypes and alleles of *ZNF676* rs412658 and *CTC1* rs3027234 in female patients with myopia and the control group.

Polymorphism	Genotype/ allele	Frequency (%)		
		Control group, n=149	Myopia group, n=73	*p*-Value
ZNF676 rs412658	**Genotype**			
	CC	52 (34.9)	31 (42.5)	0.229
	CT	76 (51)	37 (50.7)	
	TT	21 (14.1)	5 (6.8)	
	Total	149 (100)	73 (100)	
	**Allele**			
	C	180 (60.4)	99 (67.8)	0.129
	T	118 (39.6)	47 (32.2)	
CTC1 rs3027234	**Genotype**			
	CC	99 (66.4)	42 (57.5)	0.428
	CT	43 (28.9)	27 (37)	
	TT	7 (4.7)	4 (5.5)	
	Total	149 (100)	73 (100)	
	**Allele**			
	C	241 (80.9)	111 (76)	0.236
	T	57 (19.1)	35 (24)	

p-value – level of significance; differences were considered statistically significant when p<0.05.

Table S5. Binominal logistic regression analysis of *ZNF676* rs412658 and *CTC1* rs3027234 in the female control and myopia groups.

Model	Genotype/allele	OR (95% CI)	*p*-Value	AIC
***ZNF676* rs412658**				
Codominant	C/T *vs.* C/C<br>T/T *vs.* C/C	0.817 (0.451 – 1.478)<br>0.399 (0.137 – 1.167)	0.503<br>0.093	282.058
Dominant	C/T+T/T *vs.* C/C	0.726 (0.409 – 1.289)	0.274	282.017
Recessive	T/T *vs.* C/T+C/C	0.448 (0.162 – 1.241)	0.123	280.505
Overdominant	C/T *vs.* C/C+T/T	0.987 (0.564 – 1.728)	0.964	283.204
Additive	T	0.700 (0.451 – 1.089)	0.113	280.644
***CTC1* rs3027234**				
Codominant	C/T *vs.* C/C<br>T/T *vs.* C/C	1.480 (0.811 – 2.701)<br>1.347 (0.374 – 4.846)	0.201<br>0.648	283.524
Dominant	C/T+T/T *vs.* C/C	1.461 (0.822 – 2.598)	0.196	281.544
Recessive	T/T *vs.* C/T+C/C	1.176 (0.333 – 4.153)	0.801	283.143
Overdominant	C/T *vs.* C/C+T/T	1.447 (0.800 – 2.617)	0.222	281.727
Additive	T	1.319 (0.825 – 2.110)	0.248	281.882

p-value – level of significance; differences were considered statistically significant when p<0.05; OR – capability ratio; AIC – Akaike.

Table S6. Frequencies of genotypes and alleles of *ZNF676* rs412658 and *CTC1* rs3027234 in male patients with myopia and the control group.

Polymorphism	Genotype/ allele	Frequency (%)		
		Control group, n=51	Myopia group, n=27	*p*-Value
ZNF676 rs412658	**Genotype**			
	CC	25 (49)	15 (55.6)	0.845
	CT	21 (41.2)	10 (37)	
	TT	5 (9.8)	2 (7.4)	
	Total	51 (100)	27 (100)	
	**Allele**			
	C	71 (69.6)	40 (74.1)	0.558
	T	31 (30.4)	14 (25.9)	
CTC1 rs3027234	**Genotype**			
	CC	29 (56.9)	17 (63)	0.863
	CT	16 (31.4)	7 (25.9)	
	TT	6 (11.8)	3 (11.1)	
	Total	51 (100)	27 (100)	
	**Allele**			
	C	74 (72.5)	41 (75.9)	0.648
	T	28 (27.5)	13 (24.1)	

p-value – level of significance; differences were considered statistically significant when p<0.05.

Table S7. Binominal logistic regression analysis of *ZNF676* rs412658 and *CTC1* rs3027234 in the male control and myopia groups.

Model	Genotype/allele	OR (95 % CI)	*p*-Value	AIC
***ZNF676* rs412658**				
Codominant	C/T *vs.* C/C<br>T/T *vs.* C/C	0.794 (0.295 – 2.132)<br>0.667 (0.115 – 3.876)	0.647<br>0.652	104.286
Dominant	C/T+T/T *vs.* C/C	0.769 (0.301 – 1.963)	0.583	102.323
Recessive	T/T *vs.* C/T+C/C	0.736 (0.133 – 4.072)	0.725	102.498
Overdominant	C/T *vs.* C/C+T/T	0.840 (0.322 – 2.194)	0.722	102.498
Additive	T	0.807 (0.388 – 1.677)	0.565	102.289
***CTC1* rs3027234**				
Codominant	C/T *vs.* C/C<br>T/T *vs.* C/C	0.746 (0.256 – 2.178)<br>0.853 (0.188 – 3.860)	0.592<br>0.836	104.327
Dominant	C/T+T/T *vs.* C/C	0.775 (0.298 – 2.020)	0.603	102.352
Recessive	T/T *vs.* C/T+C/C	0.938 (0.215 – 4.085)	0.932	102.618
Overdominant	C/T *vs.* C/C+T/T	0.766 (0.269 – 2.176)	0.616	102.370
Additive	T	0.867 (0.437 – 1.720)	0.682	102.456

p-value – level of significance; differences were considered statistically significant when p<0.05; OR – capability ratio; AIC – Akaike.

Figure S1. Comparison of relative leukocyte telomere length and genotypes of *ZNF676* rs412658 and *CTC1* rs3027234 gene polymorphisms in female and male myopia patients.

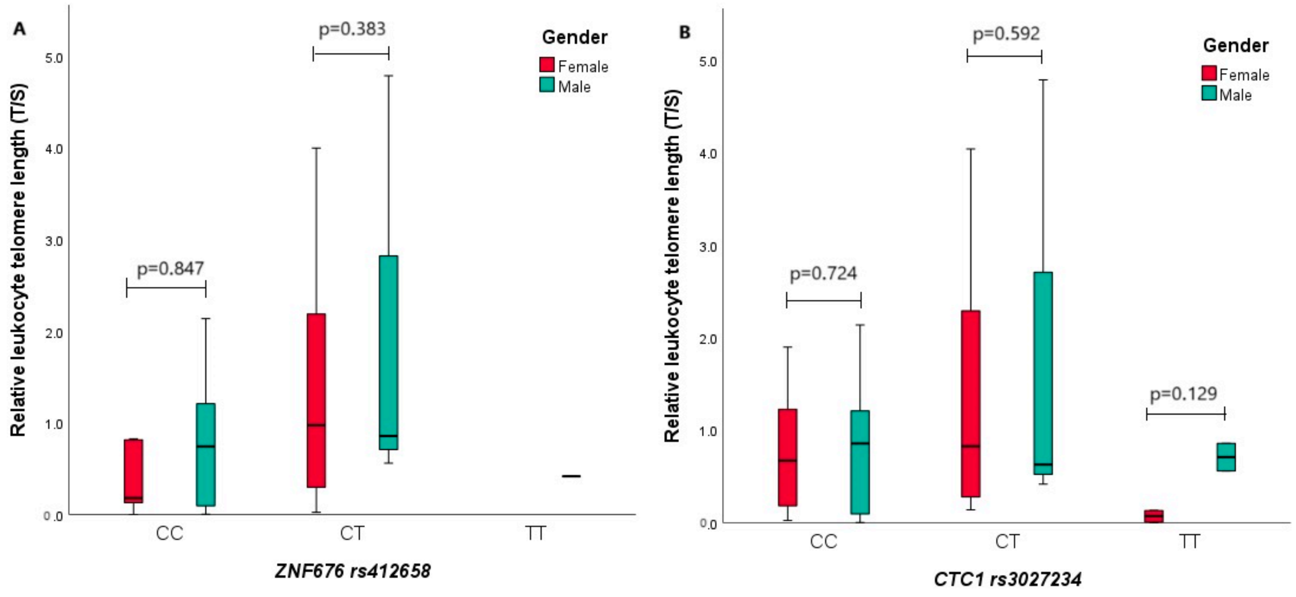

The X-axis shows the genotypes of the ZNF676 rs412658 and CTC1 rs3027234 gene polymorphisms, the Y-axis shows the relative length of the leukocyte telomeres, and the blue and green rectangles, divided by the median, represent quartiles. The lower part is the first quartile, the upper part is the third quartile; the p-value is the level of significance (differences are considered statistically significant when p<0.05). Student's t-Test was used.

Table S8. Medians of the *ZNF676* rs412658 and *CTC1* rs3027234 genotypes distributions with telomere length according to myopia degree.

Myopia degree	Median (IQR)	*p*-Value
***ZNF676* rs412658 CC genotype**		
Weak (n=11)	0.627 (0.717)	0.056*
Moderate (n=6)	0.027 (0.396)	
High (n=7)	0.528 (1.238)	
***ZNF676* rs412658 CT genotype**		
Weak (n=10)	1.758 (2.371)	0.207*
Moderate (n=4)	0.492 (1.533)	
High (n=3)	0.628 (-)	
***CTC1* rs3027234 CC genotype**		
Weak (n=8)	0.819 (1.494)	0.223*
Moderate (n=6)	0.084 (0.924)	
High (n=8)	0.737 (1.077)	
***CTC1* rs3027234 CT genotype**		
Weak (n=11)	0.825 (2.242)	-
Moderate (n=1)	-	
High (n=2)	0.462 (-)	
***CTC1* rs3027234 TT genotype**		
Weak (n=3)	0.559 (-)	0.100**
Moderate (n=3)	0.016 (-)	

p-value – level of significance; differences were considered statistically significant when p<0.05.

**Kruskal-Wallis test was used*

***Mann-Whitney U test was use*

Figure S2. *ZNF676* rs412658 genotypes associations with relative leukocyte telomere length according to myopia degree.

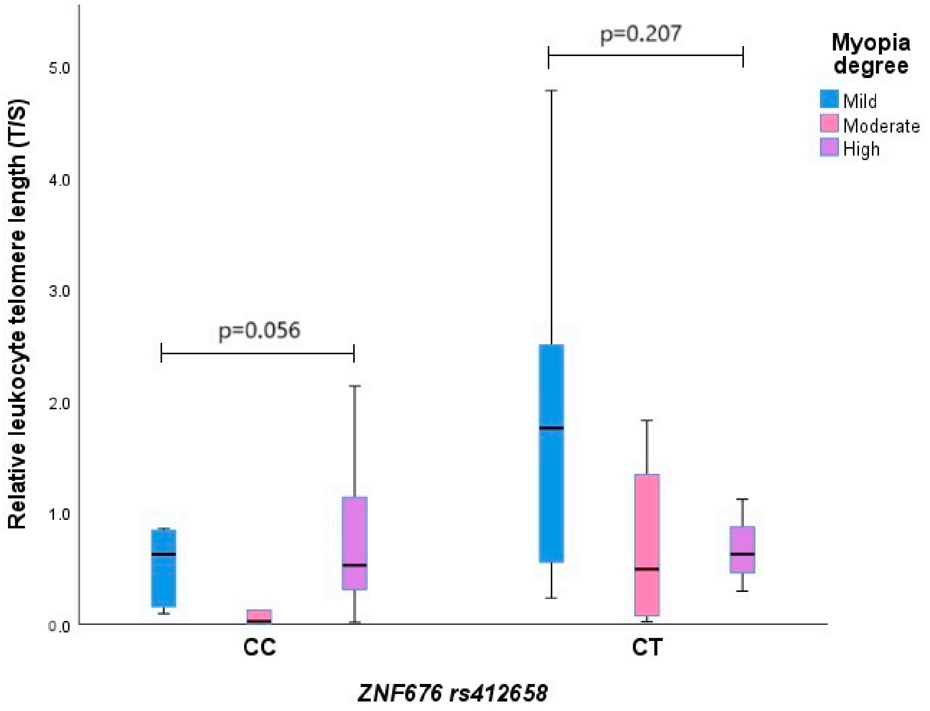

The X-axis shows the genotypes of the ZNF676 rs412658 gene polymorphisms, the Y-axis shows the relative length of the leukocyte telomeres, and the blue, pink and purple rectangles, divided by the median, represent quartiles. Myopia degree is showed according to the color of rectangles. The lower part is the first quartile, the upper part is the third quartile; the p-value is the level of significance (differences are considered statistically significant when p<0.05).

Figure S3. *CTC1* rs3027234 genotypes associations with relative leukocyte telomere length according to myopia degree.

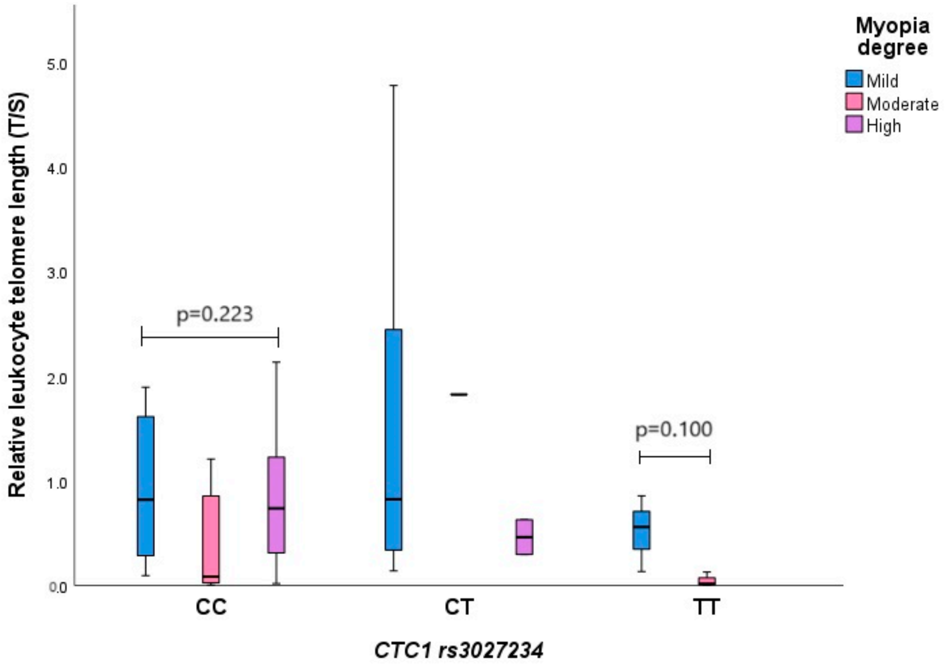

The X-axis shows the genotypes of the CTC1 rs3027234 gene polymorphisms, the Y-axis shows the relative length of the leukocyte telomeres, and the blue, pink and purple rectangles, divided by the median, represent quartiles. Myopia degree is showed according to the color of rectangles. The lower part is the first quartile, the upper part is the third quartile; the p-value is the level of significance (differences are considered statistically significant when $p<0.05$).
